# Pronunciation assessment in foreign language learning: Reliability and scoring bias in human–generative AI evaluation

**DOI:** 10.1371/journal.pone.0354603

**Published:** 2026-07-29

**Authors:** Whyunyoung Choi

**Affiliations:** Department of Educational Research, County South, Lancaster University, Lancaster, United Kingdom; University of Lahore - Raiwind Road Campus: The University of Lahore, PAKISTAN

## Abstract

This study examines the reliability and scoring bias of generative AI (Gen-AI)-based pronunciation assessment compared with human raters, addressing whether AI-generated scores can be trusted in real educational settings. Sixty students participated in a 12-week program. A total of 180 pronunciation samples were evaluated across eight subcomponents (individual phonemes, stress, rhythm, intonation, linking, reduction, fluency, and clarity) by three standardized human raters and Gen-AI using the same 7-point rubric. Quantitative analyses (intraclass correlation coefficients, paired-samples t-tests, and Pearson correlations) assessed reliability and bias, while semi-structured interviews with raters provided explanatory qualitative insights. Gen-AI demonstrated moderate reliability with human raters across most components, showing the highest agreement in fluency and the weakest in individual phonemes. However, Gen-AI consistently assigned significantly higher scores than human raters across all subcomponents. Qualitative findings revealed that discrepancies originated from Gen-AI’s limited discriminative power, systematic flaws in handling missing data, decontextualized scoring approach, lack of sensitivity to L1 interference, and inability to interpret pragmatic context. While Gen-AI cannot fully replace human expertise, it can function as a complementary tool for formative assessment and autonomous practice. A hybrid assessment model integrating Gen-AI’s efficiency with human raters’ contextual and interpretive insights is recommended for effective foreign language pronunciation education.

## 1. Introduction

Pronunciation is a core element of communication; without clear pronunciation, effective communication becomes difficult even with sufficient grammatical knowledge or vocabulary [1,2]. Pronunciation directly affects intelligibility and communication confidence [3], which in turn leads to learners’ sustained motivation for second language learning and actual communicative competence [4]. Therefore, pronunciation instruction has been consistently regarded as a crucial issue in English education.

However, pronunciation teaching in foreign language (FL) classroom settings, such as English classrooms in Korea, faces several practical constraints [5,6]. First, it is practically difficult for a single teacher to meticulously diagnose individual pronunciation and provide customized feedback to multiple learners within limited class time [7,8]. Second, many non-native FL teachers have not received sufficient professional training in pronunciation pedagogy, which makes it difficult to provide systematic pronunciation instruction [9,10]. Third, pronunciation assessment is prone to subjectivity, making it challenging to ensure consistency and reliability among evaluators [11]. Fourth, learners frequently miss opportunities for effective correction because they cannot receive immediate feedback after pronunciation practice [12].

As an innovative tool to overcome these limitations, generative artificial intelligence (Gen-AI), such as ChatGPT, has attracted increasing attention [13]. In particular, Gen-AI’s multimodal capability to process text and voice simultaneously is opening new possibilities in the field of pronunciation education [14]. Specifically, Gen-AI not only supports individualized pronunciation practice by generating customized texts suited to learners’ situations [4,13], but also provides immediate and detailed feedback [7]. Furthermore, beyond simple learning support, it performs assessment functions that systematically diagnose learners’ pronunciation, score it, and present detailed suggestions for improvement [15], demonstrating the potential to transform the paradigm of pronunciation education.

Previous studies have also reported positive results regarding the effectiveness and assessment feasibility of AI-based pronunciation learning [16,17]. However, most research has focused on technical aspects (e.g., performance validation of multimodal models or speech recognition accuracy [18,19]) and has not sufficiently addressed how reliably learners’ pronunciation can be assessed in actual classroom contexts [20]. In particular, empirical studies verifying how closely AI-based assessment aligns with human expert judgment targeting high school students in FL environments remain very limited.

Therefore, this study seeks to address the key question: “*Can AI-based pronunciation assessment be trusted in real FL classrooms?*” Specifically, it conducts a multifaceted comparative analysis of assessment reliability between Gen-AI (ChatGPT-4o) and human raters targeting Korean high school students’ English pronunciation. Through this, the study aims to identify the strengths and limitations of Gen-AI-based pronunciation assessment, explore complementary utilization strategies with human raters, and provide empirical evidence on whether Gen-AI can establish itself as a reliable tool in actual classroom instruction for pronunciation assessment.

## 2. Literature review

### 2.1. Structural limitations of traditional FL pronunciation education

Pronunciation education in traditional FL classrooms has limited effectiveness due to several structural constraints [15]. Above all, the shortage of classroom time restricts opportunities for learners to sufficiently practice pronunciation and engage in meaningful speaking activities [21]. Within an overloaded curriculum, teachers find it difficult to allocate sufficient pronunciation practice time to each learner, and particularly, high student-to-teacher ratios make individual diagnosis and customized instruction virtually impossible [22,23]. Another major issue lies in the lack of teacher expertise. Many FL teachers have not received systematic training in pronunciation instruction, which often leads to overly general or insufficiently specific feedback. Consequently, learners seldom receive the precise and systematic feedback necessary for pronunciation improvement [24,25].

Furthermore, the limitations of human assessment weaken the reliability of pronunciation education. Teachers are easily exposed to fatigue and lack of consistency, and the characteristic of relying on subjective interpretation can lead to biased judgments such as the “halo effect,” which can undermine the fairness and validity of assessment [20]. Additionally, the process of listening to, analyzing, and scoring speech samples requires considerable time and resources, making it difficult to provide immediate feedback. The delay between performance and feedback reduces the effectiveness of correction, causing learners to miss opportunities to modify their pronunciation in a timely manner [26,27]. This is particularly detrimental to pronunciation learning, where immediate correction is crucial.

### 2.2. The potential of AI in FL pronunciation education

Recent advances in AI and automatic speech recognition (ASR) technology present possibilities for complementing the structural limitations faced by traditional FL pronunciation education [28,29]. AI-based learning tools provide learners with an environment where they can learn at their own pace without temporal or spatial constraints, and offer opportunities to practice pronunciation repeatedly without fear of making mistakes [30]. In addition, speech assessment systems integrating natural language processing and big data technology enable learners to access diverse L2 pronunciation learning materials, and provide individualized diagnosis and customized feedback [31]. Through this, learners can monitor their pronunciation abilities more closely, and the specific and detailed feedback provided by AI supports substantial linguistic achievement [8]. Furthermore, unlike human raters, AI is not affected by fatigue or personal bias and can thus present more standardized and objective criteria [32–34]. Particularly in that rapid assessment and individual feedback provision are possible even with large learner populations, AI demonstrates the potential to enhance accuracy, efficiency, and consistency in pronunciation education and strengthen reliability and fairness [35,36].

### 2.3. Limitations and challenges of AI-based pronunciation assessment

Although AI technology offers several advantages for language learning, limitations that still need to be resolved exist in the context of FL pronunciation education [37]. The most important challenge is the lack of accuracy in speech recognition [38,39]. Current AI-based speech assessment systems have lower accuracy in speech recognition and transcription compared to human listeners [39] and have not secured sufficient robustness in recognizing the diverse accents or prosodic characteristics of non-native FL speakers [40]. Due to these limitations, AI may provide inaccurate or fragmentary feedback, which can lead learners with diverse linguistic backgrounds to doubt the reliability of AI feedback and negatively affect their confidence in utilizing such tools [41].

Additionally, AI systems face inherent limitations in capturing and appropriately reflecting the complexity and cultural nuances of human language [7,42]. In particular, when AI models are trained predominantly on FL samples from specific regions, there is a risk of inappropriately judging or unfavorably assessing FL variations used by non-native FL speakers [15]. This can cause systematic bias against specific learner groups and undermine the fairness and equity of assessment [37]. These technical and cultural constraints remain important challenges that must be resolved for AI-based pronunciation assessment tools to establish themselves as reliable assessment means in FL educational settings.

### 2.4. Comparative analysis of human and AI assessment

Recent studies comparing human raters and AI assessment report that the latest Gen-AI models and some commercialized automated evaluation systems (AESs) show statistically high consistency and reliability [43]. However, it remains necessary to critically examine whether such consistency truly reflects accuracy or merely results from inherent leniency bias. In fact, some AI systems exhibit systematic score inflation bias [20], suggesting that continuous validation and calibration of algorithms are essential. Additionally, the accuracy of AI assessment varies significantly depending on task type; for example, it showed high correlation with human raters in controlled tasks such as “reading aloud,” but accuracy decreased markedly in spontaneous speech assessments such as “presentation” [18].

Similar patterns have been observed across assessment domains. Gen-AI is effective in evaluating segmental features such as the accuracy of individual phonemes, but shows relatively lower accuracy in assessing suprasegmental features such as stress, rhythm, and intonation [28]. In particular, it struggles to detect stress errors caused by FL learners’ L1 interference [20]. Research findings are also conflicting regarding the quality of feedback. Some studies report that ChatGPT-4o provides more comprehensive and actionable feedback than humans [7], while other studies criticize that AI feedback is biased toward numerical scores and lacks specific explanations and corrective guidance [15,18]. Comprehensively, previous studies suggest that AI has the greatest educational value when utilized as a complementary tool that supplements human professional judgment rather than completely replacing human raters [7,20].

### 2.5. Research gap and the need for the present study

Although previous studies have highlighted the potential of Gen-AI in pronunciation education and assessment, several important research gaps still exist. First, because the emergence of Gen-AI is relatively recent, Gen-AI-based language assessment research has primarily focused on the writing domain [44–46], and studies empirically verifying the reliability of pronunciation assessment using the latest Gen-AI models are relatively scarce [7,47]. Second, while existing studies tend to focus on the learning effectiveness of Gen-AI tools, learner perceptions, or affective factors [7,8,15,28,48], studies directly comparing the quality of scores and feedback generated by Gen-AI with human experts are very limited [20]. Third, most previous studies have targeted university students or adult learners [7,8,20], and the actual FL classroom context of secondary education has been relatively overlooked. Therefore, this study aims to provide empirical evidence on the reliability and educational applicability of pronunciation assessment using Gen-AI and fill the research gap by conducting a multifaceted comparison of Gen-AI and human rater results targeting Korean high school students in an English as a foreign language (EFL) context. The research questions of this study are as follows:

**•** RQ1. What is the degree of reliability between Gen-AI and human raters in pronunciation assessment targeting high school learners in an EFL environment?
**•** RQ2. What factors cause the differences between Gen-AI and human assessment?
**•** RQ3. What are the strengths and limitations of Gen-AI assessment and human assessment as perceived by human raters?

## 3. Theoretical framework

This study adopts an ontological perspective grounded in critical realism. This is a position that views the objective reality of pronunciation performance as existing, while the process of evaluating and interpreting that performance is constructed by the epistemic characteristics of the assessment subject and social, linguistic, and technological contexts [49,50]. From this perspective, the difference between human and Gen-AI-based assessment is understood not as a simple numerical comparison, but as a constructive difference in capturing reality at different epistemic and technological levels.

Additionally, this study employs a mixed-methods approach based on pragmatist epistemology. Pragmatism views quantitative and qualitative approaches as flexibly integrable for achieving research purposes, and enables multilayered understanding of phenomena beyond the limitations of a single methodology [51]. Accordingly, this study sought to explore the reliability and educational applicability of Gen-AI-based pronunciation assessment by utilizing objective numerical evidence (quantitative evidence) and subjective perception (qualitative insight) in a complementary manner.

## 4. Methodology

### 4.1. Research design

This study adopted a one-group quasi-experimental design to compare the reliability between Gen-AI-based pronunciation assessment and human raters based on pronunciation samples collected in an authentic educational context. Additionally, a mixed-methods research design was applied to utilize quantitative and qualitative data in a complementary manner. Quantitative analysis focused on statistically verifying the reliability between Gen-AI-based pronunciation assessment and human rater results, while qualitative analysis focused on exploring the causes of these results and examining in depth the complementary characteristics of the two assessment methods.

### 4.2. Participants

The participants were 60 students recruited through convenience sampling from a girls’ high school located in Chungcheongnam-do. Recruitment was conducted over one week beginning on December 9, 2024, with 20 students evenly assigned from each grade level. All participants participated voluntarily with written consent obtained, and their English proficiency level was at B1–B2 based on Common European Framework of Reference for Languages (CEFR) standards.

### 4.3. Research procedure

This study was conducted over a total of 12 weeks. In Week 1, an orientation was held to guide participants on the study’s purpose, procedures, and Gen-AI tool usage guidelines, and a pre-assessment was conducted. The pre-assessment utilized The Rainbow Passage [52], which is widely used for English pronunciation assessment [53]. From Week 2 to Week 10, learners performed self-directed pronunciation practice using ChatGPT-4o at least three times per week, approximately 30 minutes each session, with customized scripts (see S1 Text). The researcher respected learners’ autonomy while monitoring the appropriateness of feedback when necessary. In Week 5, a mid-assessment was conducted, and in Week 11, a post-assessment was conducted, using B2- and C1- level scripts generated by Gen-AI tailored to each learner’s progress (see S2 Text).

All speech samples were evaluated using a rubric adapted by Choi [4] from Isaacs and Trofimovich [54] for EFL learner pronunciation assessment (see S3 Table). The evaluation criteria consisted of eight categories (individual phonemes, stress, rhythm, intonation, linking, reduction, fluency, and clarity), and both human raters and Gen-AI assigned 7-point Likert scale scores and descriptive feedback based on the same rubric. As students read the scripts aloud via ChatGPT-4o’s Voice Mode and provided real-time speech input, the input speech was automatically processed and transcribed by ChatGPT’s integrated speech recognition system, while the researcher simultaneously recorded the same input using a separate device (smartphone) in mp4 format under identical conditions. Independent scoring by human raters and Gen-AI was conducted based on these identical speech samples, ensuring comparability.

Human evaluation: Three experts with over 10 years of experience participated (a Korean English teacher with a Ph.D., a Korean-Canadian English teacher who completed doctoral coursework, and an American English teacher with a Master’s degree). Prior to evaluation, a standardization workshop was conducted to align interpretations and applications of the rubric. Each rater then independently listened to the audio recordings, assigned scores on a 7-point Likert scale, and provided brief descriptive feedback for each sample (see S4 Table). The average of the three raters’ scores was used as the final human score, and the intraclass correlation coefficient (ICC) was .882, indicating a high level of inter-rater reliability [55].

Gen-AI evaluation: To ensure consistency in assessment, student profiles, rubric criteria, scoring guidelines, and feedback formats were input in advance (see S2 Text). The system was configured to provide immediate evaluation and feedback on real-time speech input, and all assessments for each student were conducted within the same chat session throughout the 12-week program (see S5 Text).

**Fig 1:**
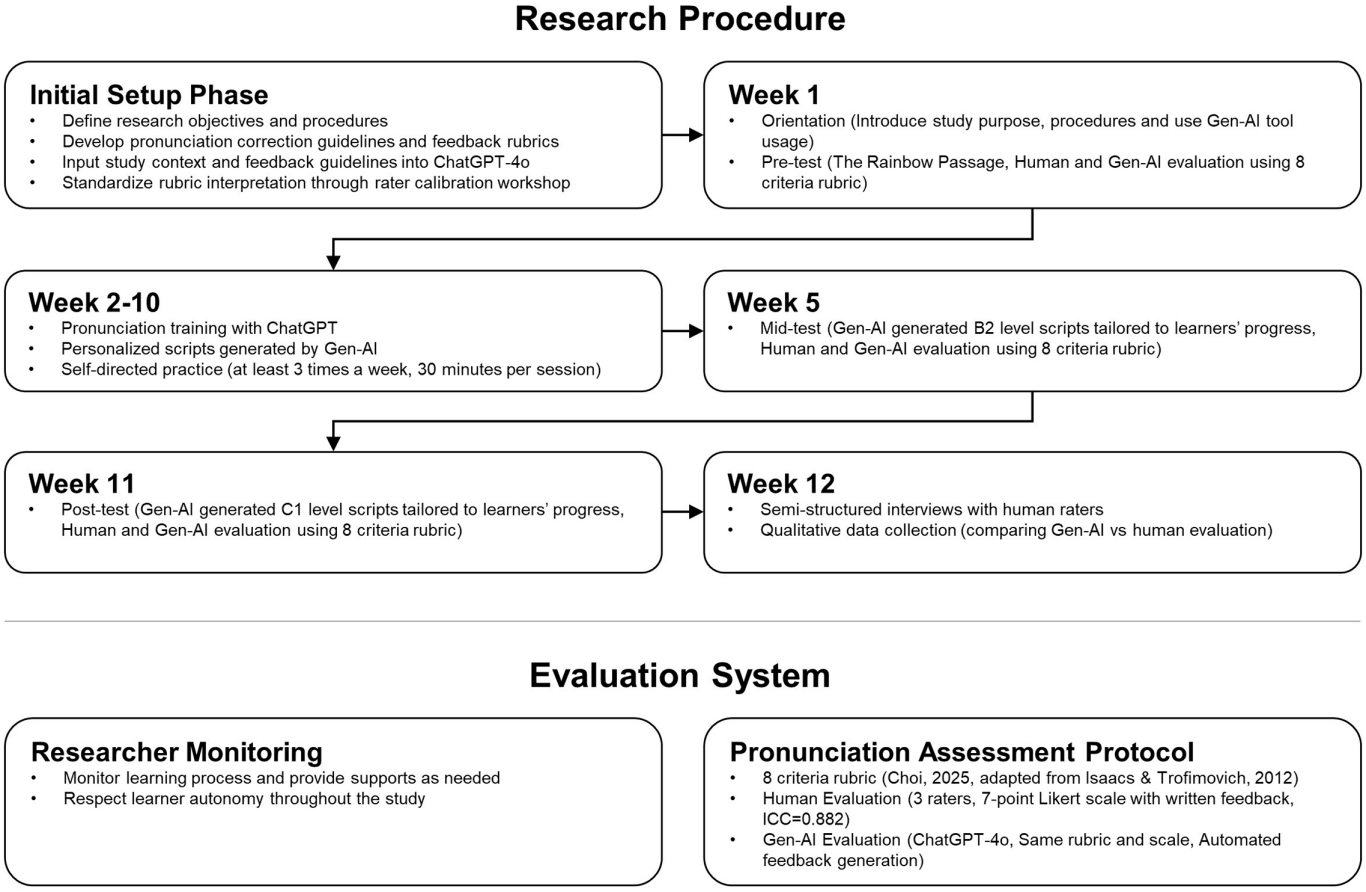
Research Procedure Flowchart.

In the final Week 12, semi-structured interviews were conducted with all human raters to collect qualitative data for comparative analysis between Gen-AI and human assessment (see Fig 1).

### 4.4. Data analysis

Quantitative data were analyzed using SPSS ver. 27. As establishing an independent ground truth for pronunciation quality is inherently difficult, the averaged scores of three expert raters who demonstrated high inter-rater reliability following a standardization workshop were used as the rubric-based expert reference criterion for comparison with Gen-AI assessment results. The reliability between Gen-AI and human raters for a total of 180 pronunciation samples was verified as follows: First, the absolute agreement between Gen-AI and human raters was measured through the intraclass correlation coefficient (ICC) using a two-way random effects model with single measures (ICC [1,2]) to evaluate overall reliability. ICC values were interpreted according to Koo and Li’s [55] guidelines: values below.50 indicate poor agreement,.50–.75 moderate,.75–.90 good, and.90 or above excellent agreement. Second, a paired samples t-test was conducted to verify the statistical significance of mean differences between Gen-AI and human raters, thereby identifying the presence of systematic bias. Effect sizes were calculated using Cohen’s d and interpreted according to Cohen’s [56] conventions: d = .2 (small), .5 (medium), and .8 (large). Third, the linear relationship between the two assessment results was analyzed through Pearson correlation coefficient to verify rank-order agreement. These analyses were performed for each of the eight pronunciation elements included in Choi’s [4] rubric.

Qualitative data were analyzed using Clarke and Braun’s [57] six-phase thematic analysis. The analysis focused on interview transcripts where human raters reviewed and discussed both their own evaluation results and those generated by the Gen-AI. Through this, the causes of quantitative analysis results were explored and the strengths and weaknesses of each assessment subject were identified. To secure the credibility of the analysis, a member checking procedure was implemented in which interview participants reviewed and confirmed the researcher’s initial coding.

### 4.5. Ethical considerations

This study was conducted in accordance with the ethical principles of the Declaration of Helsinki and approved by the Educational Research Ethics Committee, Faculty of Arts and Social Sciences, Lancaster University (Application ID: EdRes-2024–5030-EdAp-1, 29/11/2024). Written informed consent was obtained from all participants and their guardians prior to participation. Participation was voluntary, and participants were informed of their right to withdraw at any time without penalty. All data were anonymized and used solely for research purposes.

## 5. Findings

### 5.1. Quantitative results

As a result of analyzing the ICC for the eight sub-elements of pronunciation, the absolute agreement between human raters and Gen-AI assessment showed differences by item. The moderate reliability of agreement was confirmed in fluency (ICC = .637, 95% CI [.312,.786]), while individual phonemes (ICC = .210, 95% CI [–.140,.467]) remained at a poor level, and the remaining elements such as stress (.496), rhythm (.454), intonation (.430), linking (.475), reduction (.512), and clarity (.448) ranged from poor to moderate levels. Subsequently, as a result of conducting a paired samples t-test, a tendency for Gen-AI to consistently assign higher scores than human raters was confirmed across all sub-elements (*p* < .001). The difference was particularly large in individual phonemes (*ΔM* = –1.622, *d* = –1.33), intonation (*ΔM* = –1.139, *d* = –0.89), linking (*ΔM* = –1.097, *d* = –0.93), and reduction (*ΔM* = –1.206, *d* = –0.99), while it was moderate in stress (*ΔM* = –0.836, *d* = –0.69), rhythm (*ΔM* = –0.622, *d* = –0.50), clarity (*ΔM* = –0.633, *d* = –0.61), and fluency (*ΔM* = –0.711, *d* = –0.65). Finally, in the Pearson correlation analysis, significant positive correlations appeared in all elements (*p* < .01). Among these, fluency (*r* = .564), reduction (*r* = .509), and linking (*r* = .460) showed relatively high correlations, while stress (*r* = .430), rhythm (*r* = .348), intonation (*r* = .407), and clarity (*r* = .389) showed moderate levels of correlation. In contrast, individual phonemes (*r* = .271) showed low correlation, suggesting that Gen-AI assessment does not consistently align with human raters’ assessment patterns (see Table 1).

**Table 1:** Comparison of human and Gen-AI evaluations across pronunciation subcomponents.

Pronunciation Component	Human *M* (*SD*)	Gen-AI *M* (*SD*)	ICC (95% CI)	*ΔM*	*t*(179)	*d*	*r*
Individual phonemes	3.486 (1.084)	5.108 (0.934)	.210 (–.140 –.467)***	–1.622	–17.777***	–1.325	.271**
Stress	3.794 (1.231)	4.631 (0.998)	.496(.142 –.685)***	–0.836	–9.300***	–0.693	.430**
Rhythm	3.689 (1.171)	4.311 (0.969)	.454(.212 –.615)***	–0.622	–6.768***	–0.504	.348**
Intonation	3.706 (1.247)	4.844 (1.087)	.430(–.027 –.662)***	–1.139	–11.953***	–0.891	.407**
Linking	3.611 (1.201)	4.708 (1.066)	.475(–.033 –.707)***	–1.097	–12.430***	–0.926	.460**
Reduction	3.586 (1.264)	4.792 (1.200)	.512(–.057 –.745)***	–1.206	–13.233***	–0.986	.509**
Fluency	3.611 (1.251)	4.322 (1.046)	.637(.312 –.786)***	–0.711	–8.767***	–0.653	.564**
Clarity	4.111 (1.086)	4.744 (0.712)	.448(.155 –.628)***	–0.633	–8.156***	–0.608	.389**

Note. ICC = Intraclass Correlation Coefficient (Absolute agreement); *ΔM* = Mean Difference (Gen-AI – Human); *d* = Cohen’s *d*; *r* = Pearson Correlation Coefficient.

*** *p* < .001; ** *p* < .01.

Viewed comprehensively, Gen-AI secured a certain level of reliability and correlation with human raters in pronunciation assessment. However, it showed a lenient tendency to assign generally higher scores than humans. In particular, while fluency showed relatively high reliability and correlation—implying potential for practical application—lower reliability in individual phonemes and other detailed pronunciation components indicated clear limitations in Gen-AI’s capacity for fine-grained diagnostic evaluation.

### 5.2. Qualitative results

As a result of interviews with human raters, various perceptions were revealed regarding the causes of discrepancies between Gen-AI and human evaluations. This qualitative analysis provides important interpretive insight into the quantitative findings, particularly explaining why Gen-AI consistently awarded higher scores and why reliability varied across pronunciation sub-elements.

#### 5.2.1. Raters’ perceptions of causes of discrepancies between Gen-AI and human assessment.

**5.2.1.1. Lack of Discriminative Power and Structural Flaws in Handling Missing Data:** Human raters perceived that the lenient assessment tendency of Gen-AI revealed in the quantitative results stemmed from a lack of discriminative power—its inability to distinguish among learner proficiency levels. Gen-AI assigned appropriate scores to learners with excellent pronunciation but tended to give similar scores to those with weaker pronunciation, failing to capture fine-grained qualitative distinctions in pronunciation ability. This appears to result from an algorithmic limitation that processes all utterances above a certain level as “good.” Raters analyzed that, based on the sufficiently secured inter-rater reliability (ICC = .882), the main cause of score differences lies in Gen-AI’s lack of discriminative power and structural limitations rather than human inconsistency.

*“(ChatGPT) gives almost students’ scores in the 6-point range once they reach a certain level. But all three of us gave that same student scores in the 4-point range.” [Rater A, July 14]*

A systematic flaw in handling missing data was identified as a specific cause of this lack of discriminative power. When learners read unfamiliar scripts generated by Gen-AI, they often omitted or mispronounced them. While human raters consistently treated such omissions as clear errors and deducted points accordingly, Gen-AI excluded unpronounced words from its scoring scope, generating evaluations based only on the portions that were spoken. This is interpreted as a result of Gen-AI employing a narrowed evaluation approach that analyzes only the technical accuracy of input speech signals without considering task completion. This flaw convincingly explains the low ICC and correlation coefficient (*r* value) in the individual phonemes, as well as the overall inflation phenomenon of Gen-AI scores confirmed in the t-test.

*“Even though the script was generated by the Gen-AI itself, if a student skipped parts (did not read as non-existent), the Gen-AI simply ignored them and scored only what was read, the scores inevitably came out higher.” [Rater B, July 15]*

**5.2.1.2. Decontextualized Evaluation and Lack of Sensitivity to L1 Interference Patterns:** Raters attributed another source of discrepancy between Gen-AI and human evaluations to decontextualized and mechanical-like assessment approach. Human raters recognized that learners’ individual phoneme pronunciation had not changed substantially during the research period, and maintained consistency by tolerating minor errors that did not impede communication in an FL context. In contrast, the Gen-AI was viewed as excessively focused on the mechanical perfection of isolated speech samples, undermining the consistency and validity of assessment. In particular, due to Gen-AI’s tendency to score each phoneme independently as if comparing it with an ideal native-FL (English) speaker model and correct answer, it regarded even natural variations in connected speech—such as assimilation and coarticulation—as errors.

*“The student’s /l–r/ pronunciation stayed about the same throughout the three months. We knew that and scored consistently, but the AI gave high scores one day and low scores another... It seemed to only look at the recording quality of that moment.” [Rater C, July 23]*

*“The student naturally pronounced ‘and ensure’ as [ænɪnˈʃʊr], but the ChatGPT deducted points (in the pronunciation rating). This naturally occurs in fast speech, but it seemed to treat only the dictionary form as the ‘correct’ one.” [Rater B, July 22]*

This decontextualized assessment was embodied in the Gen-AI’s lack of sensitivity to L1 interference patterns. Human raters consistently deducted points for Korean L1 interference patterns—such as confusion between /l/ and /r/ in word-medial positions, failure to distinguish between /b/ and /p/ or between /p/ and /f/—within a cross-linguistic context, but Gen-AI failed to sensitively capture such transfer patterns. Stress errors in polysyllabic words (e.g., collaboration) were also pointed out as vulnerable points that Gen-AI failed to accurately identify.

*“The student pronounced ‘collaboration’ as coLLAboration, but ChatGPT didn’t catch this. It’s a common error pattern among Korean learners.” [Rater C, July 16]*

**5.2.1.3. Segmental Assessment of Suprasegmental Features and Failure to Recognize Pragmatic Context:** Meanwhile, regarding the reason why statistically significant differences were confirmed in suprasegmental features such as stress, intonation, linking, and reduction, raters found the cause in Gen-AI’s segmental scoring method. While humans judge holistically the ‘musicality’ of speech—that is, the overall impression and meaning conveyance power created by the combination of stress, rhythm, and intonation—Gen-AI was interpreted as failing to reflect the organic interaction of these elements by taking an approach of evaluating each element independently and simply summing them. Additionally, Gen-AI did not properly recognize phonological changes such as assimilation that naturally occur during linking or reduction processes, showing a tendency to regard normal variations in fast speech as errors.

*“Pronunciation isn’t about individual notes—it’s the whole melody. But Gen-AI seems to evaluate as if it’s listening to each instrument separately in an orchestra, it can’t see the whole picture.” [Rater A, July 21]*

Finally, the absence of pragmatic context recognition also appeared distinctly. Raters pointed out that even when students used natural intonation appropriate to the situation while reading sentences like “That’s right, Dr. Kim!” in dialogues, Gen-AI failed to reflect such emotional expression or genre-specific discourse context in its scoring criteria. This demonstrates a fundamental limitation where Gen-AI is sensitive to acoustic patterns but cannot capture the pragmatic or situational appropriateness of utterances.

*“In a dialogue, the student used very natural intonation, but the ChatGPT gave a lower score. I think it’s because the intonation didn’t match the ‘standard’ pattern... It shows the AI has no sense of context at all.” [Rater A, July 14]*

#### 5.2.2. Perceptions of strengths and limitations of Gen-AI and human assessment.

Human raters cited accessibility, immediacy, and psychological stability as strengths of Gen-AI-based assessment. Gen-AI provides feedback without temporal or spatial constraints and can continuously perform assessment without fatigue even with repeated requests, being evaluated as having high practicality as a learning tool that supports learners’ autonomous and repetitive practice. The ability to secure a high degree of formal consistency by quickly providing item-by-item comments based on the same rubric was also mentioned as a strength. Additionally, the fact that it could reduce the anxiety accompanying presentation in front of teachers or peers and enables learners to continue speech practice in a psychologically safe environment was also positively evaluated.

*“The fact that students can practice anytime, anywhere and get feedback right away is definitely a big advantage. Gen-AI continuously assesses all eight rubric items without fatigue. We can’t possibly do that ourselves.” [Rater C, July 16]*

However, despite these strengths, the limitations of Gen-AI assessment were also clear. First, due to lack of contextual understanding, Gen-AI failed to reflect FL learners’ L1 interference patterns or individual learning processes, and showed a tendency to overestimate actual ability by assigning excessively lenient scores to lower-level learners due to lack of discriminative power. A more serious problem was the lack of reliability in feedback. In some cases, Gen-AI presented pronunciation information incorrectly, identified correctly pronounced parts as errors, and even hallucination phenomena were observed where it generated content not in the script. Such instances were pointed out as a serious limitation that could cause learners to internalize misleading information.

*“Gen-AI told the student to put stress on the first syllable of ‘produce’, but the student had already pronounced it correctly. There were even cases where it gave feedback on words the student didn’t say and weren’t in the script at all.” [Rater B, July 22]*

Additionally, the lack of actionability in Gen-AI feedback was pointed out. Although superficially appearing rich and detailed, it lacked practical examples of specific locations or methods for intonation adjustment and could not replace teacher talk—a teaching strategy that helps learners intuitively recognize errors through exaggerated or slow demonstration pronunciation. Finally, structural limitations were confirmed where discriminative power weakens particularly in prosodic and pragmatic-based elements such as intonation, linking, and reduction, because it fails to reflect affective and pragmatic cues such as learner anxiety or discourse context.

*“Gen-AI gives a lot of feedback, but it never really tells students how to fix their pronunciation. When we show it exaggeratedly, students immediately go ‘Ah!’ and realize... And even when a student captured the right nuance (in a dialogue), Gen-AI just said ‘nonstandard intonation’.” [Rater B, July 15].*

In contrast, human assessment showed strengths in the ability to reflect linguistic and cultural context, consider learners’ affective and situational variables, and evaluate pronunciation holistically. Raters interpreted Korean learners’ typical L1 interference patterns (/l–r/, /b–p/, /p–f/ confusion) and stress errors in polysyllabic words within a cross-linguistic context, and comprehensively judged the ‘musicality’ of speech—how stress, rhythm, and intonation interacted organically—as well as the appropriateness of intent, emotion, and situation. They also reflected affective and situational contextual factors that Gen-AI cannot capture, such as distinguishing between cases where fluency temporarily drops due to learner nervousness and actual language ability deficiency, and considering learners’ metacognitive responses that adjust language use according to task type (e.g., dialogues vs. presentations).

*“One student was usually fluent but stuttered because she was nervous that day. We considered it, but the Gen-AI just labeled it as ‘low fluency.’ And we can recognize that students adjust their speech style differently for dialogues versus presentations, but Gen-AI can’t see that.” [Rater C, July 23]*

Furthermore, human raters demonstrated sophisticated evaluative intuition and expertise by emphasizing the systematicity and repetitive patterns of errors rather than simple error frequency, and judging the balance between consistency and accuracy throughout the utterance. They also performed corrective and instructional roles difficult for Gen-AI to replace by helping learners intuitively recognize their own errors through teacher talk strategies and providing specific, contextualized feedback based on experiential judgment.

*“Everyone makes mistakes once or twice. What’s important is whether the same error keeps repeating. We judge based on those patterns, but Gen-AI just seems to count the number of mistakes.” [Rater A, July 21]*

However, limitations of human assessment were also clearly revealed. Raters pointed out that it is difficult to maintain objectivity of judgment when repeatedly listening to the same passage, and fatigue accumulation from prolonged assessment can lead to decreased judgment consistency. They also mentioned that due to temporal and physical constraints, it is difficult to provide sufficient feedback to all learners within limited class time, resulting in limitations in immediacy, accessibility, and scale.

*“Honestly, if I listen to the same recording multiple times, my judgment gets blurred. When I’m assessing the 20th person in the afternoon, concentration drops... and there’s no time to give sufficient feedback to all 20 students during class.” [Rater A, July 21]*

Additionally, it was acknowledged as a realistic limitation that external variables—such as assessment settings (e.g., face-to-face vs. recording) or task instruction methods (“just read it” vs. “read it as if acting”)—could influence outcomes.

*“The feeling is different when evaluating face-to-face versus listening to a recording, and student responses differ when saying ‘just read it’ versus ‘read it as if acting’... all these become variables.” [Rater C, July 16]*

Based on these observations, raters proposed a complementary integration model combining Gen-AI and human assessment. They agreed that Gen-AI is suitable for providing immediate, formally aligned primary diagnosis and repetitive practice feedback for large-scale learners, while human raters are effective as in-depth assessment providing contextualized feedback by comprehensively considering L1 interference patterns, prosodic and pragmatic elements, and affective variables. Therefore, a hybrid model combining Gen-AI’s efficiency with human interpretive power, that is, combining primary Gen-AI screening with secondary in-depth human feedback, was agreed upon as the most practical and efficient approach in actual classroom settings.

*“Gen-AI handles the first round—giving instant, consistent feedback to hundreds of students or supporting repetitive practice—and then we provide the second, deeper layer of feedback. This combination would work best.” [Rater B, July 15]*

## 6. Discussion

This study empirically analyzed the differences between Gen-AI-based pronunciation assessment and human raters in a Korean EFL high school context and explored the causes in depth, thereby bridging the gap between the technical void in existing automated evaluation systems (AESs) research and the practical needs of educational settings, and expanding the technology-centered discourse of Gen-AI-based assessment research to educational and epistemological dimensions.

The findings showed that Gen-AI exhibited a certain level of agreement with human raters, but overall revealed lenient score assignment and limitations in contextual understanding. This partially aligns with previous studies [8,28] that AI provides consistency and efficiency in assessment, but demonstrates that such efficiency does not necessarily guarantee fairness and validity in assessment. Notably, this study empirically identified a narrowed evaluation pattern in Gen-AI responses, in which learners’ utterances appeared to be processed primarily at the level of acoustic signals without reflecting task completeness. This pattern was associated with score inflation and reduced discriminative power, as discussed by Chen and Sun [20]. These findings suggest a difference in evaluative orientation, where Gen-AI responses tended to prioritize acoustic accuracy (technical measurement) over task completeness (educational assessment), extending the discussion of contextual limitations raised by X. Liu et al. [37] and Mao et al. [42]. These findings suggest that for Gen-AI-based assessment tools to secure pedagogical validity in educational settings, fundamental redesign of evaluation scope and criteria is necessary.

Gen-AI’s decontextualized assessment and its limited sensitivity to systematic L1 interference patterns further elaborates the discussion of non-native speaker pronunciation bias presented by Emara and Shaker [40] and Zou et al. [15]. The observed tendency for Gen-AI to process each utterance as an independent event, insufficiently reflect the temporal consistency of learner pronunciation, and treat natural phonological changes or intonation variations as errors may be interpreted as suggesting epistemic limitations related to contextual interpretation: a potential possibility not to insufficiently reflect the diversity of FL. This suggests that the phenomenon previous research labeled as “bias” may be better understood as an issue potentially related to the absence of contextual validity rather than a simple technical problem, and raises the need for alignment with the communication-centered philosophy of pronunciation pedagogy [1,2] that Gen-AI-based assessment should consider sociolinguistic appropriateness alongside phonetic accuracy.

In the analysis by pronunciation sub-element, results contrary to Abdelhalim and Alsehibany’s [28] report that AI shows higher accuracy in segmental features than suprasegmental features. In this study, while low reliability and correlation were confirmed in individual phonemes, moderate levels of reliability appeared in stress, rhythm, and intonation, and interestingly, relatively high agreement was confirmed only in fluency. This inconsistency ultimately comes down to the question not simply of “what is measured” but “how it is measured.” Gen-AI reveals a paradigm difference between analytic and holistic scoring by taking a segmental assessment approach that scores each element independently and simply sums them, rather than judging suprasegmental elements as holistic “musicality.” While human raters perceive utterances as a gestalt and comprehensively assess overall impression and communicative effect, Gen-AI separates each element and reduces it to measurable variables. This approach reconfirms, as pointed out by Chen and Sun [20], Emara and Shaker [40], and X. Liu et al. [37], that AI does not sufficiently reflect the organic interaction of prosodic elements and pragmatic context. Additionally, Gen-AI demonstrated a tendency to process even contextually appropriate intonation as “non-standard” when pragmatic factors such as discourse situation, emotional expression, and genre characteristics were not reflected. This pattern suggests that while Gen-AI demonstrates sensitivity to capturing acoustic patterns, it has limitations in adequately reflecting the sociocultural meaning of utterances, indicating that human raters’ holistic judgment ability remains important.

These findings also carry important theoretical implications. From the perspective of critical realism and pragmatism—the philosophical foundations of this research—the differences between human and Gen-AI assessment can be interpreted not merely as numerical discrepancies but as reflecting operations at different epistemic layers [49,50]. While learners’ pronunciation performance constitutes an objective reality, the processes of evaluation and interpretation are shaped by the epistemic characteristics of the assessor and by social, linguistic, and technological contexts. Gen-AI tends to capture reality primarily within the empirical domain of acoustic signals, relying on physical characteristics. In contrast, human raters may interpret beyond the observable level by considering deeper factors that can influence speech performance—such as learners’ L1 background, learning trajectory, pragmatic intentions, and affective states—which are not directly detectable but shape speech performance. This epistemological difference suggests that Gen-AI-based assessment may not fully replace human assessment but can instead function as a complementary tool that illuminate different layers of reality. In this regard, the qualitative analysis in this study should be understood not as an attempt to directly uncover the internal computational mechanisms of the Gen-AI system itself, but as an approach that interprets observed assessment behavior in authentic educational contexts from pedagogical and epistemological perspectives. Such interpretive insights provide a contextual and ecological understanding that technical model analysis alone cannot sufficiently capture.

In conclusion, by integratively illuminating the reliability and limitations of Gen-AI-based assessment, this study proposes that Gen-AI should serve as a complementary collaborative partner rather than completely replacing human raters. This perspective empirically supports the need for a “hybrid assessment model” emphasized by Chen and Sun [20] and Li et al. [7], and presents the direction for constructing a new assessment paradigm that combines Gen-AI’s technical precision with human interpretive expertise.

## 7. Implications

The findings of this study demonstrate the potential for Gen-AI to enhance the efficiency of pronunciation education and assessment, while also suggesting that human rater expertise remains essential. Based on these results, the following educational and practical implications are proposed.

### 7.1. Establishing a Gen-AI–based formative assessment model

Gen-AI should be utilized as a formative assessment tool rather than summative assessment. The lenient assessment tendency and lack of discriminative power revealed in this study can undermine fairness in high-stakes assessments such as end-of-semester evaluations or qualification exams. However, Gen-AI’s immediacy and accessibility are effective for learners to continuously monitor their pronunciation and identify directions for improvement during the learning process. Therefore, teachers should employ Gen-AI for formative assessment purposes such as daily pronunciation practice, self-diagnosis, and learning progress tracking, but must combine professional judgment of human raters in summative assessments such as end-of-semester grading or promotion decisions. Specifically, a hybrid feedback model can be constructed where learners autonomously use Gen-AI-based pronunciation feedback before and after assignments for self-directed practice, after which teachers provide supplementary instruction focusing on major error patterns. This approach can be a realistic way to support individual learner’s pronunciation improvement even in large-scale classes. Additionally, non-face-to-face practice with Gen-AI can reduce the anxiety felt in front of teachers or peers, while promoting fluency through repetitive learning.

### 7.2. Strengthening teachers’ mediating role

Considering the limitations in the reliability of Gen-AI feedback, teachers’ pre- and post-checking procedures should be essential. The hallucination phenomenon confirmed in this study—cases where Gen-AI provides feedback on words learners did not pronounce or points out correct pronunciation as errors—poses a risk of learners internalizing incorrect information and potentially fossilizing inaccurate pronunciation patterns. This raises a significant educational and ethical concern, as learners with lower proficiency—who are less likely to possess the metalinguistic awareness needed to critically evaluate AI-generated feedback—may be particularly vulnerable to internalizing inaccurate linguistic models without question. Therefore, teachers should instruct learners not to accept Gen-AI feedback uncritically, but to develop an attitude of checking its validity by themselves and consulting the teacher whenever they feel uncertain. In practice, learners can be required to capture and submit screenshots of any Gen-AI feedback that they find unclear or questionable, and request teacher confirmation; such a procedure can be codified as part of classroom policy.

Additionally, as confirmed in this study, teacher talk (the use of exaggerated model pronunciation) or intuitive visualizations plays a crucial role in helping learners instantly perceive and correct their errors. Therefore, while using Gen-AI feedback as supplementary material, corrective activities where teachers should also conduct direct modeling activities to highlight contrasts and guide accurate correction should be combined. Furthermore, for elements where pragmatic context is important (e.g., intonation, linking, and reduction), teachers should explicitly explain discourse situation, emotional tone, and genre characteristics and guide practice within authentic communicative contexts, rather than relying solely on Gen-AI’s mechanical feedback.

### 7.3. Enhancing teacher competence and directions for technological advancement

At the teacher-education level, there is a need to strengthen AI-human hybrid assessment literacy. In a situation where many non-native FL teachers have not received systematic training in pronunciation instruction, Gen-AI can serve as a supplementary tool to complement teachers’ lack of expertise. However, effective use requires teachers to have a solid understanding of how Gen-AI operates, its strengths and limitations, and reliability verification methods. Therefore, educational authorities and teacher-training institutions should develop practical, competency-based training programs including (1) methods for using various Gen-AI-based pronunciation assessment tools and designing effective prompts, (2) criteria for evaluating the reliability of Gen-AI feedback and identifying hallucination phenomena, (3) strategies for applying Gen-AI to specific pronunciation components and determining appropriate timing for human intervention, (4) hybrid assessment model design and implementation cases sharing, and (5) pedagogical approaches for developing learners’ Gen-AI literacy. Through such training, teachers will be equipped with the competence to utilize Gen-AI critically and strategically, rather than blindly trusting or unconditionally rejecting it.

At the technical level, continuous improvement of Gen-AI algorithms and development of FL learner-specific models are necessary to mitigate the limitations identified in this study—systematic flaws in handling missing data, failure to recognize L1 interference patterns, and absence of pragmatic context recognition. Particularly considering that current Gen-AI models are mainly trained on native FL speaker pronunciation data, it is urgent to expand the training corpus to include diverse non-native speech data (such as that of Korean L1 speakers) and to embed cultural-linguistic context–sensitive criteria into the algorithm. Additionally, there is a need to develop holistic scoring algorithms that comprehensively assess the organic interaction of elements, moving away from the current segmental approach of independently scoring and simply summing. Achieving this goal, close collaboration between AI developers and language education experts is essential.

## 8. Conclusion

This study empirically compared the reliability between Gen-AI-based pronunciation assessment and human raters targeting Korean EFL high school students, and explored the causes of these differences quantitatively and qualitatively, thereby identifying the educational applicability and limitations of Gen-AI-assisted pronunciation assessment. Quantitative results showed that Gen-AI exhibited relatively high reliability in fluency assessment, but demonstrated a lenient scoring tendency to consistently assign higher scores than human raters in most pronunciation elements. Qualitative findings further confirmed that these differences stemmed from Gen-AI’s narrowed evaluation approach, lack of discriminative power, decontextualized assessment approach, failure to recognize L1 interference patterns, and inability to interpret pragmatic context. In particular, Gen-AI failed to capture the organic interaction of prosodic elements due to a segmental approach of independently scoring and simply summing stress, rhythm, and intonation rather than judging them holistically, and feedback reliability issues such as hallucination phenomena and lack of actionability were also revealed. In contrast, human raters demonstrated professional intuition to holistically interpret utterances and provide contextualized feedback by comprehensively reflecting cross-linguistic context, pragmatic and affective factors. However, human raters also showed limitations in immediacy and scale due to temporal and physical constraints. These results suggest that Gen-AI cannot fully replace human raters; rather, the most effective model for actual FL classrooms is a complementary framework in which Gen-AI supports formative assessment and repetitive practice, while humans raters provide in-depth and contextually grounded feedback.

Nevertheless, this study has several limitations. First, because it targeted only a single Gen-AI model (ChatGPT-4o), there are constraints in generalizing findings to other Gen-AI systems. Therefore, the findings of this study should be interpreted as limited to general-purpose large language model (LLM)-based assessment systems such as ChatGPT-4o, rather than Gen-AI in general. Since comparative analysis among other LLMs such as Google Gemini and Claude, or various AI-based specialized pronunciation assessment systems was not performed, it remains unclear whether the observed limitations are specific to ChatGPT-4o, reflect common characteristics of general-purpose LLMs, or represent broader constraints of current Gen-AI technology. Moreover, because LLMs may exhibit non-stationarity due to updates and performance drift over time, the findings of this study need to be interpreted as limited to the model state at a specific point in time. Second, research participants were limited to 60 students from a single girls’ high school located in Chungcheongnam-do, which restricted diversity across regions, gender, and FL proficiency levels. In particular, since the study participants were at CEFR B1–B2 level, it was not confirmed how reliability between Gen-AI and human raters differs in pronunciation assessment of beginner or advanced learners. Third, this study applied the same 7-point Likert scale used by human raters to Gen-AI assessment to ensure direct comparability. However, this fine-grained numerical scale may not fully align with the evaluation characteristics of generative AI systems. In addition, although this study used the averaged scores of three trained human expert raters with high inter-rater reliability as the rubric-based reference criterion, these cannot be regarded as an absolute standard for pronunciation accuracy. Accordingly, the score differences and lenient scoring tendencies identified in this study should be interpreted not as direct evidence of absolute inaccuracy, but as differences observed in comparison with expert human assessment. Fourth, as the research period was limited to 12 weeks, it was difficult to confirm long-term learning effects or cumulative impacts of Gen-AI feedback. Longitudinal tracking was not conducted on whether learners experience pronunciation improvement through continuous interaction with Gen-AI, or whether repeated exposure to inaccurate feedback may lead to reinforcement of incorrect pronunciation habits. Fifth, the one-group design and repeated interaction with the same Gen-AI system may have introduced potential testing effects, maturation effects, or alignment bias. However, since this study focused on comparing cross-sectional agreement and systematic bias between Gen-AI and human raters using pooled data across all assessment phases, and on identifying the underlying causes through qualitative analysis, rather than tracking individual learners’ longitudinal changes, the influence of these factors is likely limited.

Building upon these limitations, future research should expand in the following directions: First, through comparative research among various Gen-AI models—particularly general-purpose LLM–based evaluators—and professional pronunciation assessment systems, the strengths and limitations of each tool should be systematically analyzed, and criteria for selecting the most appropriate AI tool according to specific pronunciation elements or learner levels should be established. Additionally, future research should verify how model version updates affect assessment consistency to establish more stable evaluation criteria. Furthermore, future studies might investigate the technical dimensions of Gen-AI scoring behavior—including prompt sensitivity, transcription accuracy, and run-to-run variability—to complement the pedagogical insights offered by applied educational research. Second, by verifying the reliability and fairness of Gen-AI-based assessment targeting various learner groups—beginner, intermediate, and advanced levels, as well as learners with diverse L1 backgrounds and gender—it should be confirmed whether Gen-AI-based assessment tools operate equitably for all learners. In particular, directions for algorithm improvement to mitigate Gen-AI’s bias problem regarding non-native FL accents and speaker characteristics (e.g., gender-related pitch differences) can be presented. Third, AI-optimized scale designs, such as binary or threshold-based classifications, should be explored as they may better suit the evaluative mechanisms of generative AI in pronunciation assessment. Fourth, through longitudinal studies, the long-term effects and cumulative impacts of Gen-AI-based pronunciation feedback on learner pronunciation improvement—both positive learning effects and potential side effects—should be evaluated in a balanced manner. Fifth, future studies should consider separating the practice tool from the evaluation system to minimize potential alignment bias. Sixth, through collaborative research between AI developers and language education experts, pronunciation assessment models specialized for FL learners should be developed, and technical improvement measures including L1 interference pattern recognition, pragmatic context reflection, and holistic scoring algorithms should be specified. Finally, through action research that explores the potential of Gen-AI-human hybrid assessment models in authentic classroom settings, practical guidelines should be developed for teachers and learners to effectively utilize Gen-AI. Collectively, these follow-up studies will establish a practical and theoretical foundation for Gen-AI to function as a complementary partner with human raters in FL pronunciation education.

## Supporting information

S1 Text Example of personalized scripts for pronunciation practice (CEFR B2 level dialogue script generated by ChatGPT-4o). (PDF)

S2 Text ChatGPT-4o prompt used for pronunciation assessment and personalized script generation. (PDF)

S3 Table Analytical rubric profile for pronunciation evaluation (adapted from Choi, 2025). (PDF)

S4 Table Sample of human raters’ evaluation scores and feedback. (PDF)

S5 Text Sample of Gen-AI (ChatGPT-4o) evaluation and real-time feedback. (PDF)
