# Supplementary material for: Pronunciation assessment in foreign language learning: Reliability and scoring bias in human–generative AI evaluation
Source: PLoS One. 2026 Jul 29;21(7):e0354603. doi: 10.1371/journal.pone.0354603 (PMC13419193; doi:10.1371/journal.pone.0354603)
Supplement: S1 Text — (PDF) [file pone.0354603.s001.pdf]

Supporting Information (S1 Text):

Example of Personalized Script for Pronunciation Practice (CEFR B2 Level Dialogue Script, Generated by ChatGPT-4o)

Topic: Ethical Considerations in Pharmaceutical Research

Participants: [redacted] (Pharmaceutical Researcher), Dr. Park (Research Director)

Director: [redacted], as you know, ethical considerations are crucial in our research. How do we ensure our studies meet ethical standards?

Researcher: We follow strict guidelines set by regulatory bodies and ethics committees. These guidelines help us protect the rights and well-being of participants.

Director: That’s correct. What specific measures do we take to protect participants in our clinical trials?

Researcher: We obtain informed consent from all participants, ensuring they understand the risks involved. We also monitor their health closely throughout the trial.

Director: Exactly. Informed consent is a key component. How do we handle situations where the potential benefits of a drug might outweigh the risks?

Researcher: In such cases, we have to weigh the benefits against the risks carefully. If the potential benefits are significant, we might proceed, but with increased monitoring and safeguards.

Director: That’s right. We must always prioritize the safety and rights of participants. What’s your view on the ethical challenges we face in developing new drugs?

Researcher: I believe it’s our responsibility to balance innovation with ethics. We need to push the boundaries of science, but never at the expense of participants’ well-being.

Director: Well said. It’s a delicate balance, but it’s essential for maintaining trust in our research.

Researcher: I’ll keep that in mind, Dr. Park. Ensuring ethical integrity is just as important as achieving scientific breakthroughs.

Director: Absolutely. Keep up the good work, and remember that ethical considerations should guide every decision you make in your research.

Practice Points:

1. **Word Pronunciation**: Focus on clear pronunciation of words like “ethical considerations,” “informed consent,” “safeguards,” “regulatory bodies,” and “well-being.”
2. **Stress**: Use correct stress in phrases like “protect the rights,” “weigh the benefits against the risks,” and “ethical integrity” to emphasize meaning.
3. **Rhythm**: Practice maintaining consistent rhythm in longer sentences like “We need to push the boundaries of science, but never at the expense of participants’ well-being.”

4. **Linking**: Practice natural linking of words in phrases like “ethical considerations are crucial” and “increased monitoring and safeguards.”
5. **Intonation**: Practice adjusting intonation naturally to emphasize key points in sentences like “It’s a delicate balance, but it’s essential for maintaining trust in our research.”
