# Supplementary material for: Pronunciation assessment in foreign language learning: Reliability and scoring bias in human–generative AI evaluation
Source: PLoS One. 2026 Jul 29;21(7):e0354603. doi: 10.1371/journal.pone.0354603 (PMC13419193; doi:10.1371/journal.pone.0354603)
Supplement: S3 Table — (PDF) [file pone.0354603.s003.pdf]

Supporting Information (S3 Table):

Analytical Rubric Profile for Pronunciation Evaluation (Choi, 2025)

Pronunciation Rating Profile		
Student Name:		Audio number:
Instructor:		Date:
Record Score	Score Range	
1. Segmental Error (Individual Sound Production): Evaluates the accuracy of individual phoneme pronunciation, including vowels and consonants.		
	6-7	All phonemes are pronounced accurately, sounding clear and natural.
	4-5	Most phonemes are pronounced correctly, but there are occasional errors with complex sounds (e.g., /θ/, /ʒ/).
	2-3	Some phonemes are correct, but repeated errors hinder understanding.
	1	Frequent errors make words difficult to understand.
Comments:		
2. Stress: Assesses the correct placement of stress within words and sentences to clarify meaning.		
	6-7	Stress is consistently accurate, making speech clear and natural.
	4-5	Stress is generally correct, with minor errors in longer sentences.
	2-3	Stress is occasionally correct but often misplaced, affecting meaning.
	1	Stress is incorrectly placed or absent, making speech unclear.
Comments:		
3. Rhythm: Measures the natural flow of speech through the coordination of stressed and unstressed syllables.		
	6-7	Stress and unstress are well-balanced, creating a smooth and natural rhythm.
	4-5	Rhythm is mostly natural but occasionally disrupted.
	2-3	Rhythm is inconsistent, with unnatural pauses and breaks.
	1	Speech lacks rhythm entirely, sounding robotic or disjointed.
Comments:		
4. Intonation: Evaluates the use of rising and falling tones to convey meaning and engage the listener.		
	6-7	Intonation fully enhances meaning and keeps the listener engaged.
	4-5	Intonation is mostly appropriate but occasionally awkward.
	2-3	Intonation patterns are sporadic or fail to convey meaning effectively.
	1	Intonation is monotonous or inappropriate, making speech robotic.
Comments:		
5. Linking: Assesses the smooth connection between words in speech to ensure flow.		
	6-7	All words are naturally linked, creating seamless and smooth speech.
	4-5	Most words are linked appropriately, with occasional disconnections.
	2-3	Some linking occurs, but most words are pronounced separately.
	1	Words are disconnected, making speech choppy and unnatural.
Comments:		
6. Reduction: Evaluates the use of reduced forms (e.g., "and the" as /ən ðə/) to ensure naturalness.		

	6-7	Reductions are applied consistently and naturally across all appropriate contexts.
	4-5	Reductions are mostly accurate, with minor inconsistencies or errors.
	2-3	Reductions are attempted but inconsistently or inaccurately applied.
	1	Reductions are rarely used or applied inappropriately, making speech sound unnatural.
Comments:		
7. Fluency: Measures the ability to speak smoothly and continuously without frequent hesitations.		
	6-7	Speech is natural and smooth, making it easy for listeners to follow.
	4-5	Speech is mostly fluent, with occasional pauses or hesitations.
	2-3	Speech flow is disrupted by frequent pauses or slow delivery.
	1	Speech is halting, with many interruptions or repetitions.
Comments:		
8. Clarity: Assesses how understandable the speech is to the listener.		
	6-7	Speech is consistently clear, even in complex sentences.
	4-5	Speech is generally clear, but complex phrases may reduce clarity.
	2-3	Speech lacks clarity, with some words or sentences difficult to understand.
	1	Speech is very unclear, making it hard for listeners to comprehend.
Comments:		
Total Score		Comments:

Student Name:		Audio number:
Category	Score	Comments
1. Segmental Error		
2. Stress		
3. Rhythm		
4. Intonation		
5. Linking		
6. Reduction		
7. Fluency		
8. Clarity		
Total Comments:		
