# Supplementary material for: Pronunciation assessment in foreign language learning: Reliability and scoring bias in human–generative AI evaluation
Source: PLoS One. 2026 Jul 29;21(7):e0354603. doi: 10.1371/journal.pone.0354603 (PMC13419193; doi:10.1371/journal.pone.0354603)
Supplement: S4 Table — (PDF) [file pone.0354603.s004.pdf]

Supporting Information (S4 Table):

Sample of Human Raters' Evaluation and Feedback

Pronunciation Rating Profile (Rater 1)

Student Name:		Audio number: 5
Category	Score	Comments
1. Segmental Error	4	th sounds become s sound "ethical", "throughout", added syllabes "risk-suh" "safeguards" occassional unclear consonant sounds, slight mumbling.
2. Stress	4	Stress placement a bit random, not always on key words.
3. Rhythm	4	Flow a bit uneven, needs smoother connection between words.
4. Intonation	3	Still sounds robotic and flat, little variation.
5. Linking	2	Words mostly separated, not much linking.
6. Reduction	2	Few or no reductions, sounds too careful.
7. Fluency	3	Pace slow and hesitant, long pauses between phrases.
8. Clarity	4	Mostly clear but some blurred endings and mumbling.
Total Comments: Speech sounds careful but low-energy. Needs stronger articulation and more natural flow. Perhaps she needs to open her mouth wider when she speaks and practice short connected phrases. Student must build confidence and practice clearer pronunciation.		

Pronunciation Rating Profile (Rater 2)

Student Name:		Audio number: 5
Category	Score	Comments
1. Segmental Error	4	/θ/ → /s/ again, some missing consonants, "benefits" bit unclear. kind of mumbled.
2. Stress	3	Important words not stressed, overall pretty flat.
3. Rhythm	3	Choppy flow, too many small stops mid-sentence.
4. Intonation	3	Flat tone, almost same pitch whole time.
5. Linking	3	Words sound separate, not much connection.
6. Reduction	3	No reduction forms, sounds like reading word by word.
7. Fluency	3	slow and a bit hesitant, needs more confidence.
8. Clarity	4	Mostly clear but low volume, consonants fade sometimes.
Speech feels careful but lacks energy and confidence. Needs stronger voice and smoother flow. Practicing short, linked phrases will help build fluency.		

Pronunciation Rating Profile (Rater 3)

Student Name:		Audio number: 5
Category	Score	Comments
1. Segmental Error	4	th → s sound "ethical," some unclear endings, but overall understandable.
2. Stress	3	Main words not clear. flat tone.
3. Rhythm	3	A few pauses between words, not smooth.
4. Intonation	3	Tone rather flat, could use more variation.
5. Linking	2	Not much linking yet, words sound separate.
6. Reduction	2	Sounds like reading carefully.
7. Fluency	3	Slow and hesitant, but mostly steady.
8. Clarity	4	Understandable but quiet voice
Total Comments: The performance feels too cautious and quiet. To sound more natural, she should try reading aloud with more confidence and smoother connection between words. Speaking with more confidence will make a big difference.		
