# Supplementary material for: Pronunciation assessment in foreign language learning: Reliability and scoring bias in human–generative AI evaluation
Source: PLoS One. 2026 Jul 29;21(7):e0354603. doi: 10.1371/journal.pone.0354603 (PMC13419193; doi:10.1371/journal.pone.0354603)
Supplement: S5 Text — (PDF) [file pone.0354603.s005.pdf]

Supporting Information (S5 Text):

Sample of Gen-AI (ChatGPT-4o) Evaluation and Real-time Feedback

Student Name: **(Audio No. 5)**

1. Segmental Sounds (out of 7 points / 6)

Strengths: You paid close attention to pronunciation in key terms like *“ethical considerations,” “informed consent,”* and *“safeguards.”* It was clear that you tried to pronounce each sound carefully, even in difficult words. For example, the /k/ and /s/ sounds in *“considerations”* and the /w/ and /l/ sounds in *“well-being”* were distinct. This kind of clear sound production makes it easy for listeners to understand you.

Areas to Improve: Some words still sounded a bit unclear. For example, in *“participants,”* the /p/ and /s/ sounds were a little weak, and the /r/ in *“regulatory”* was not clearly produced. These small mistakes can blur meaning, especially in academic contexts. To improve, try repeating those words slowly and focus on making each sound clear and separate.

---

2. Stress (out of 7 points / 5)

Strengths: You used stress effectively to highlight key words and phrases. For example, in *“protect the rights,”* you placed stress on *“protect”* and *“rights,”* which helped emphasize the message. Also, in *“ethical integrity,”* your stress pattern showed the importance of the phrase well.

Areas to Improve: Some words needed clearer stress. For instance, in *“flexibility,”* the first syllable should be stressed more strongly. Missing stress can make sentences sound weaker and reduce clarity. Practice identifying which parts of a word should be stressed and repeat them until it sounds natural and strong.

---

3. Rhythm (out of 7 points / 5)

Strengths: You kept a fairly steady rhythm in long sentences. For example, in *“We need to push the boundaries of science, but never at the expense of participants’ well-being,”* your pace was smooth and steady, which made the message easy to follow.

Areas to Improve: Some parts sounded a bit rushed, such as *“increased monitoring and safeguards.”* When rhythm breaks, it’s harder for listeners to catch meaning. Try reading long sentences slowly at first, focusing on keeping an even pace before speeding up.

4. Intonation (out of 7 points / 4)

Strengths: You used intonation well to emphasize meaning in some sentences. For example, in *"It's a delicate balance, but it's essential for maintaining trust in our research,"* your pitch rose naturally on *"delicate balance"* and *"essential."* This helped convey the importance of the message.

Areas to Improve: In some sentences, your tone was a bit flat, like in *"We must always prioritize the safety and rights of participants."* Try adding more rise and fall in your tone to make your speech sound more expressive and engaging.

---

5. Linking (out of 7 points / 4)

Strengths: There was some smooth connection between words, especially between consonants and vowels, such as in *"ethical considerations are crucial."* This kind of linking helps your speech flow more naturally.

Areas to Improve: In *"increased monitoring and safeguards,"* the words sounded slightly disconnected. Try practicing transitions between words—especially between ending consonants and beginning vowels—to make your speech smoother.

---

6. Fluency (out of 7 points / 5)

Strengths: Overall, your speech was fluent and consistent. Even in longer sentences like *"We follow strict guidelines set by regulatory bodies and ethics committees,"* you maintained a steady pace and didn't pause too often.

Areas to Improve: At times, your speed changed slightly, especially in longer or more complex phrases. Try to keep the same rhythm throughout the whole sentence by reading slowly first, then gradually increasing speed while staying smooth.

---

7. Clarity (out of 7 points / 6)

Strengths: Your pronunciation was clear in most sentences, and your effort to make the content understandable was noticeable. In words like *"ethical integrity,"* your clear pronunciation made your message easy to grasp.

Areas to Improve: Some words, such as *"regulatory,"* sounded a bit blurred. Try practicing difficult words several times to keep every sound sharp and easy to recognize.

Overall Comments

, your reading was very good overall. You handled complex vocabulary and long sentences confidently, and your pronunciation was clear enough for the listener to follow easily. A bit more work on pronunciation detail and rhythm would make your delivery even stronger. Keep your strengths, and keep polishing the small parts—you're definitely on the right track!
